# Ultra-performance liquid chromatography-quadrupole\time-of- flight mass spectrometry with multivariate statistical analysis for exploring potential chemical markers to distinguish between raw and processed *Rheum palmatum*

**DOI:** 10.1186/1472-6882-14-302

**Published:** 2014-08-16

**Authors:** Zenghui Wang, Dongmei Wang, Sihao Zheng, Labin Wu, Linfang Huang, Shilin Chen

**Affiliations:** Institute of Medicinal Medicinal plant Development, Chinese Academy of Medical Sciences & Peking Union Medical College, Beijing, 100193 China; Institute of Chinese Materia Medica, China Academy of Chinese Medical Sciences, Beijing, 100193 China

**Keywords:** Ultra-performance liquid chromatography-quadrupole\time-of-flight mass spectromet- ry, Multivariate statistical analysis, *Rheum palmatum*, Herb processing, Chemical markers

## Abstract

**Background:**

The long term use of *Rheum palmatum* for the treatment of diseases associated with chronic hepatitis and renal failure can lead to liver and kidney damage. To reduce the toxicity of *R. palmatum* and alleviate any symptoms of decanta and celialgia, the raw material has been subjected to a specific process prior to its use for hundreds of years. Despite its extensive use in medicine, very little is currently known about the nature of the components present in this material in terms of their efficacy and overall toxicity, and the effect that processing has on the levels of these components in the processed material. The aim of this investigation was to explore potential differences in the chemical markers between batches of raw and processed *R. palmatum* and to develop a deeper understanding of the underlying mechanisms responsible for the enhanced efficacy and reduced toxicity of the processed material.

**Methods:**

Raw and processed *R. palmatum* samples were analyzed by ultra-performance liquid chromatography-quadrupole time-of-flight mass spectrometry (UPLC/Q-TOF-MS) coupled with multivariate statistical analysis using principal component analysis (PCA) and orthogonal partial least square discriminant analysis (OPLS-DA).

**Results:**

The emodin-8-O-glucoside, emodin-O-glucoside, catechin-glucopyranoside, gallic acid-3-O-glucoside, torachrysone, and chrysophanol dimethyl ether were rapidly explored as representative markers to distinguish for the first time between the raw and processed *R. palmatum* material. Among the potential chemical markers, Emodin-8-O-glucoside and gallic acid-3-O-glucoside were determined to be the best markers for the raw and processed *R. palmatum.*

**Conclusion:**

UPLC/Q-TOF-MS with multivariate statistical analysis represents an efficient method for exploring the chemical markers in the raw and processed *R. palmatum* material, as well as investigating the mechanisms associated with the processing, quality control, and safe application of *R. palmatum*.

## Background

Processing is an important part of traditional Chinese medicine (TCM), and most of the processes involved in TCM were developed almost 5,000 years ago, along with a number of processing technology theories and methods, such as frying with sand or oil, sautéing with rice wine or wheat bran, steaming with water or rice wine, and braising with rice wine or licorice liquids [1]. According to the theories of TCM, the main purposes of herb processing are to increase potency, reduce toxicity, and alter the effectiveness of the raw materials. The major mechanisms underlying herb processing are predominantly related to changes in the chemical composition and/or activity of the components in herbs [2].

**Figure 1:**
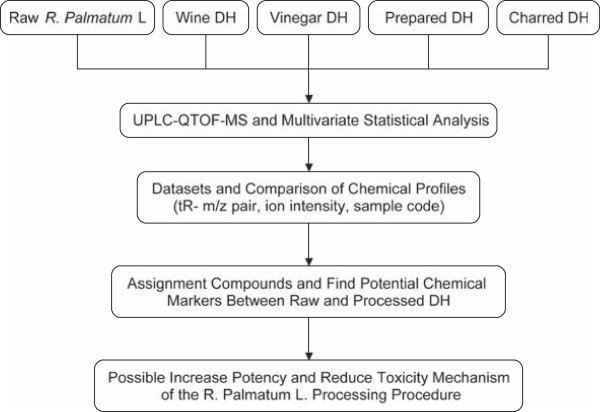
**Strategy proposed for rapidly exploring potential chemical markers for discrimination of raw and processed** ***R. palmatum*** **by UPLC–QTOF-MS coupled with multivariate statistical analysis.**

*R. palmatum*, which is known as Da-huang (DH) in Chinese, is officially listed in the Chinese Pharmacopoeia [3] and is widely used as a TCM for alleviating the symptoms of fever, moistening aridity, purging fire, and detoxifying toxicosis [4]. *R. palmatum* contains a variety of different components, including anthraquinones, dianthrones, stilbenes, anthocyanins, flavonoids, polyphenols, organic acids, and chromones [5]. Among these materials, several anthraquinone derivatives, including emodin, chrysophanol, rhein, aloe-emodin, and physcion, as well as their corresponding glucosides, have been identified as important bioactive components, and reported to exhibit a variety of pharmacological effects, such as purgative [6], anti-inflammatory [7], anticancer [8], nephric protection [9], hepatic protection [10], antimicrobial and hemostasis activities [11, 12]. Several reports have appeared in the literature, however, suggesting that the rhubarb components of *R. palmatum* have an adverse impact on liver and kidney function, as well as causing gastrointestinal reactions [13–15], and some of the anthraquinone and tannin compounds present in *R. palmatum* have been reported to be toxic [16]. *R. palmatum* has been subjected to the standard processing technologies used in TCM reduce the toxicity of the raw material, as well as alleviating any symptoms of decanta and celialgia, and the resulting material has been used in clinical practice for hundreds of years *R. palmatum* wine, prepared *R. palmatum*, and charred *R. palmatum* have been well documented in the Chinese Pharmacopoeia as therapeutic materials [4], whereas the use of vinegar *R. palmatum* has been recorded in the standardized processing of traditional Chinese medicine (version 1988) [17]. Potential chemical markers and mechanisms of increased effect and decreased toxicity of *R. palmatum* are yet to be identified and understood. In this study, we have proposed an experimental scheme of screening the markers and identifying raw and processed *R. palmatum* (Figure 1).

Ultra-performance liquid chromatography-quadrupole time-of-flight mass spectrometry (UPLC-QTOF-MS) is a newly developed technique that provides rapid and efficient access to detailed information pertaining to the nature of specific components within complex multicomponent mixtures. UPLC-QTOF-MS has been widely used in various fields for the analysis of a broad range of different materials, and has been used to particularly good effect for metabolite analysis and the identification of the complex compounds found in TCMs [18]. We previously reported the use of UPLC-QTOF-MS in conjunction with multivariate statistical analysis to discriminate between raw and processed samples of *Coptis chinensis* and *Eriobotrya japonica* [19–21]. This technique has several significant advantages over traditional phytochemical methods for identifying chemical markers, in that it avoids the requirement for time-consuming extraction, isolation, purification, and identification processes. Herein, we describe the development of an effective UPLC–QTOF-MS method that is capable of distinguishing between raw and processed *R. palmatum* (i.e., raw wine *R. palmatum*, vinegar *R. palmatum*, prepared *R. palmatum*, and charred *R. palmatum*) using principal component analysis (PCA) and orthogonal partial least square discriminant analysis (OPLS-DA). To the best of our knowledge, this method represents the first reported account of an analytical method capable of differentiating between raw and processed *R. palmatum*. Several chemical markers were screened and tentatively identified in batches of the raw and processed materials between each processed sample. The results of this study have provided a deeper understanding of the underlying mechanisms responsible for the increased efficacy and decreased toxicity of the processed material. This approach therefore represents a useful tool for distinguishing between raw and processed *R. palmatum*.

## Methods

### Chemicals, solvents, and samples

UPLC-grade acetonitrile, methanol, and formic acid were purchased from Fisher Scientific Co. (Fair Lawn, MA, USA). All of the aqueous solutions used in the current study were prepared using ultrapure water produced by a Milli-Q system (18.2 MΩ, Millipore, Billerica, MA, USA).

*R. palmatum* was collected from Gansu Minxian (Gansu, China) on the November 20, 2012. The botanical materials were identified by Professor Lin Yulin, and voucher specimens were deposited at the Institute of Medicinal Plant Development, Chinese Academy of Medical Sciences, Beijing, China. The detail of the samples’ vouchers lists as sdh1-3 of Raw DH (R), dht1-3 of Charred DH (C), shudh1-3 of Prepared DH (P), jdh1-3 of Wine DH (W), cdh1-3 of Vinegar DH (V).

### Liquid chromatography

Two microliter samples were analyzed on a 2.1 × 100 mm ACQUITY™ 1.7 μm BEH C18 column (Waters, Milford, MA, USA), which was maintained at 40°C using an Waters ACQUITY™ UPLC system. The mobile phases consisted of (A) water containing 0.1< (w/w) formic acid and (B) methanol. The UPLC elution conditions were optimized as follows: linear gradient from 5 to 15< B (0 to 2.0 min), 15 to 20< B (2.0 to 3.0 min), and 20 to 25< B (3 to 4 min), where it was held for 1 min before being increased from 25 to 30< B (5 to 6 min), 30 to 40< B (6 to 7 min), 40 to 55< B (7 to 8 min), 55 to 65< B (8 to 9 min), 65 to 85< B (9 to 10 min), 85 to 95< B (10 to 11 min), and 95 to 5< B (11 to 12 min), where it was then held for 3 min. The flow rate was set at 0.35 mL/min, and the column and autosampler were maintained at 40 and 5°C, respectively. Each wash cycle consisted of 200 μl of the strong solvent (80< ACN in water - v/v) and 600 μL of the weak solvent (30< ACN in water – v/v). The scan range for PDA detector system was set at 190 to 400 nm. All of the experiments were performed in triplicate.

### Mass spectrometry

MS analysis was performed on a Q-TOF premier mass spectrometer (Waters Micromass Technologies, Manchester, UK), which was operated in the negative ion mode using electrospray ionization. The capillary and cone voltages were set to 3000 and 35 V, respectively. The nebulization gas was maintained at a flow rate of 800 L/h and a temperature of 450°C. The cone gas was maintained at a flow rate of 50 L/h, and the source temperature was set to 120°C. MS data were collected for *m/z* values in the range of 50 to 1,200 Da with a scan time of 0.1 s and an inter-scan delay of 0.01 s over an analysis time of a 15 min. The [M–H]^−^ ion of leucine-enkephalin was seen at *m/z* 556.2771, with a concentration of 0.5 ng/μL in the negative ion mode. Argon was employed as the collision gas at a pressure of 7.066 × 10^−3^ Pa. All of the MS data were collected using the LockSpray system to ensure mass accuracy and reproducibility.

### Sample preparation

*R. palmatum* samples were processed according to the methods described in the 2010 edition of the Chinese Pharmacopoeia. Raw *R. palmatum* (R) were cut into 2- to 4-mm-thick slices or pieces and then dried. Wine *R. palmatum* (W) was prepared with rice wine (20< w/w). Vinegar *R. palmatum* (V) was prepared with vinegar (20< w/w). Prepared *R. palmatum* (P) was prepared with rice wine (20< w/w) and then steamed until it was black in appearance on both its inside and outside. Charred *R. palmatum* (C) was prepared by stir frying *R. palmatum* until it was black/brown in color on the outside and burnt-brown or black/brown in color on the inside. All of the samples were milled into powders, and individual portion of the powdered samples (0.150 g) were dissolved in methanol. The resulting samples were then extracted for 30 min using an ultrasonic cleaner in a water bath (45°C). The extracts were centrifuged at 12,000 rpm for 20 min, and the supernatant was injected directly into the UPLC-Q/TOF system for analysis.

### Establishment of an in-house library and peak assignment

Data pertaining to the different components of *R. palmatum* were collected from various different databases, including PubMed for the U.S. National Library of Medicine and the National Institute of Health, SciFinder Scholar for the American Chemical Society, and the Chinese National Knowledge Infrastructure of Tsinghua University. The collected data were summarized in a Microsoft Office Excel table to establish an in-house library, which included the name, molecular formula, UV maximum wavelength, chemical structure, and reference of each known compound. The “Find” function in Microsoft Office Excel was used to match the empirical molecular formula with that of published compounds within the library. The empirical molecular formula was deduced from and shortlisted by comparing the accurately measured mass value to the exact mass value of putative deprotonated molecular ions [M–H]^−^ at a mass accuracy of less than 5 ppm.

### Multivariate data processing

The UPLC-MS data of all determined samples were analyzed using the MarkerLynx software (Waters) to identify potential discriminatory chemical markers and allow for the raw and processed DH to be subjected to some measure of quality control. For data collection, the method parameters were set as follows: retention time in the range of 0 to 15 min, mass in the range of 50 to 1200 Da, and noise elimination level set at 5. For data analysis, a list composed of the identities of the detected peaks was generated using retention time (t_R_)–mass data (*m/z*) pairs as the identifier for each peak. An arbitrary ID was assigned to each of these t_R_–*m/z* pairs based on their order of elution from the UPLC system. The ion intensity for each detected peak was normalized against the sum of the peak intensities within that sample. Ion identification was based on the t_R_ and *m/z* values. Pareto scaling method was used to generate the PCA plot. The resulting three-dimensional data comprising the peak number (t_R_–m/z pair), sample name, and ion intensity were analyzed by PCA and OPLS-DA.

## Results and discussion

### Chromatographic conditions of UPLC

Several different mobile phase systems were evaluated in the current study, including the use of an organic phase (i.e., acetonitrile and methanol) with a variety of different aqueous phases (i.e., water, water containing formic acid, water containing triethylamine, as well as water containing formic acid and ammonium). The results of an extensive period of evaluation revealed that a mixture of acetonitrile and water (containing 0.1< formic acid) was the most suitable mobile phase. The gradient elution profile was optimized with respect to the separation of the major peaks and, under the optimized chromatographic conditions, the major components in *R. palmatum* could be well separated and detected within 15 min.

### UPLC-QTOF-MS chemical analysis

**Figure 2:**
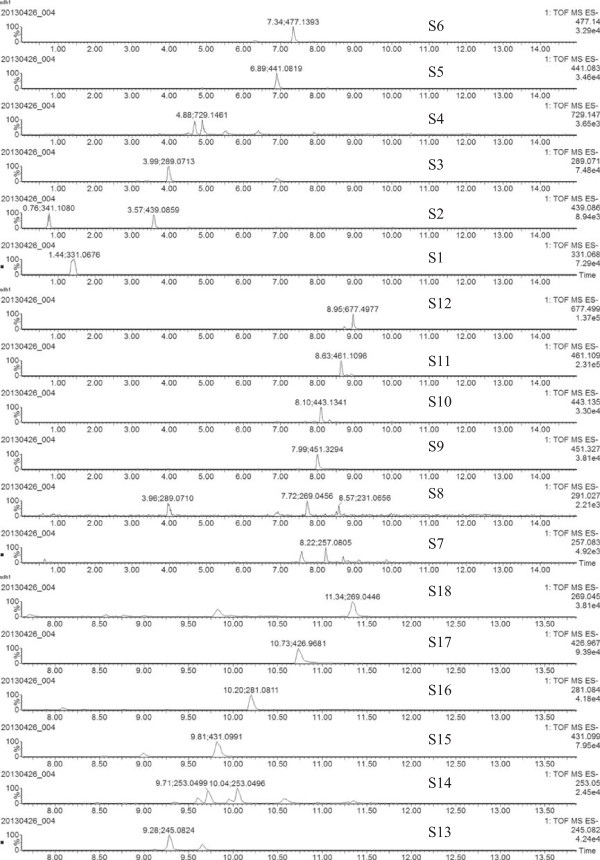
**Extracted ion chromatograms (EIC) of 18 components for** ***R. Palmatum*** **.** The peaks labeling coincide with Table 1.

18 compounds were tentatively identified based on their fragment ions (Figure 2) as well as a comparison with data from the literature [22–25]. All of these compounds have been previously reported to be present in *R. palmatum* and are listed in Table 1.

**Table 1:** **Tentative compounds in** ***R. Palmatum*** **by UPLC-QTOF/MS in negative ion**

Peak no.	t_R_(min)	m/z experimental	m/z calculated	Tentative compounds	Molecular formula	Mass error (ppm)	Reference
S1	1.43	331.0667	331.0667	Gallic acid-3-O-glucoside	C13H15O10	0	[22]
S2	3.57	439.0883	439.0883	Aloe-emodin-glucoside derivate	C21H19O10	−1.3	[22]
S3	3.96	289.0707	289.0712	Catechin	C15H13O6	−1.7	[22]
S4	4.88	729.1470	729.1456	Catechin dimer derivate	C40H69O8	1.9	[22]
S5	6.86	441.0825	441.0822	Epicatechin-3-O-gallate	C22H17O10	0.7	[22]
S6	7.33	477.1397	477.1397	Cinnamyl-galloyl-glucoside derivative	C23H25O11	0	[23]
S7	7.50	257.0827	257.0814	Emodin-O-glucoside	C15H13O4	5.1	[23]
S8	7.71	269.0450	269.0450	Aloe-emodin	C15H10O5	0	[24, 25]
S9	7.99	451.3296	451.3271	Catechin-glucopyranoside	C21H24O11	5.5	[22]
S10	8.08	443.1345	443.1345	Catechin-glucopyranoside derivative	C23H23O9	−1.7	[23]
S11	8.62	461.1085	461.1084	Cinnamyl-galloyl-glucoside derivative	C23H25O11	0.2	[23]
S12	8.95	677.4994	677.4993	Catechin dimer derivate	C40H69O8	0.3	[22]
S13	9.28	245.0824	245.0814	Torachrysone	C14H14O4	0	[23]
S14	9.71	253.0499	253.0501	Chrysophanol	C15H9O4	−0.8	[24, 25]
S15	9.81	431.0988	431.0978	Emodin-8-O-glucoside	C21H19O10	2.3	[23]
S16	10.20	281.0814	281.0811	Chrysophanol dimethyl ether	C17H14O4	−1.1	[25]
S17	10.73	426.9665	426.9691	Rhein-1-O-(O-acetyl)-glucoside	C4H11O23	−6.1	[23]
S18	11.34	269.0446	269.0450	Emodin	C15H10O5	−1.5	[24, 25]

### Confirmation of TCM processing theories with raw and processed DH

**Figure 3:**
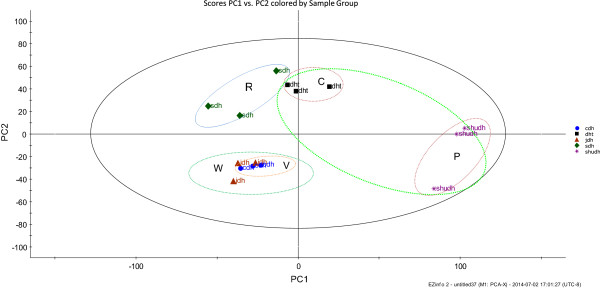
**PCA generated by the Pareto of raw and processed** ***R. Palmatum.*** R: Raw DH (sdh) C: Charred (dht) DH P: Prepared DH (hudh) W: Wine DH (jdh) V: Vinegar DH (cdh).

The results of the PCA of the raw and processed *R. palmatum* are shown in Figure 3. These results show that the raw *R. palmatum*, wine *R. palmatum*, vinegar *R. palmatum*, prepared *R. palmatum*, and charred *R. palmatum* samples were divided into five main clusters in the PCA score plot. The division of these data into clusters effectively indicated that use of different processing methods could significantly alter the composition of compounds within the different materials, and this separation could therefore be representative of their multiple pharmacological effects. According to the PCA, charred *R. palmatum* (C) clustered in the upper right region, whereas prepared *R. palmatum* (P) clustered into the bottom right region, In contrast, wine *R. palmatum* (W) and vinegar *R. palmatum* (V) clustered into the bottom left region of the PCA. The prepared *R. palmatum* (P) was clustered far from the middle region, which indicated that remarkable chemical changes had occurred during the processing of this material. The wine *R. palmatum* (W) and vinegar *R. palmatum* (V) clusters were close to each other, which indicated that similar chemical changes had occurred during their processing. The results of this experiment therefore clearly demonstrated the importance of the processing method to the chemical composition of the finished material.

### Multivariate statistical analysis and exploring chemical markers

**Figure 4:**
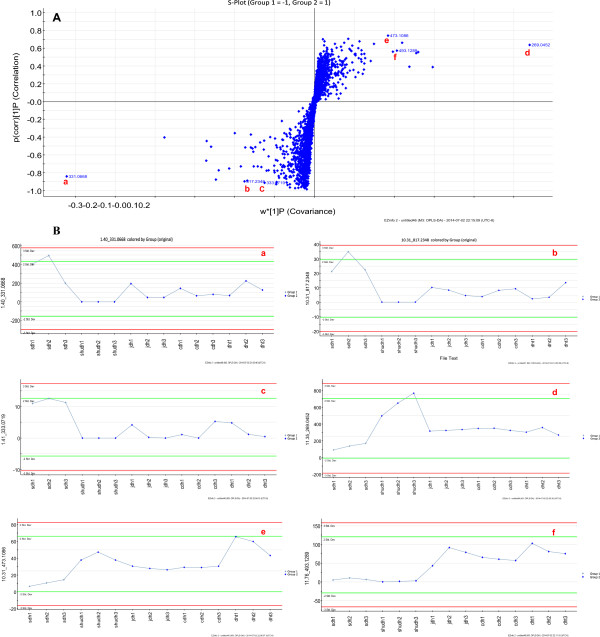
**OPLS-DA/S-plot (A) and selected ion intensity trend plots (B) of raw and processed** ***R. Palmatum.*** Group1: Raw DH Group2: Processed DH.

**Figure 5:**
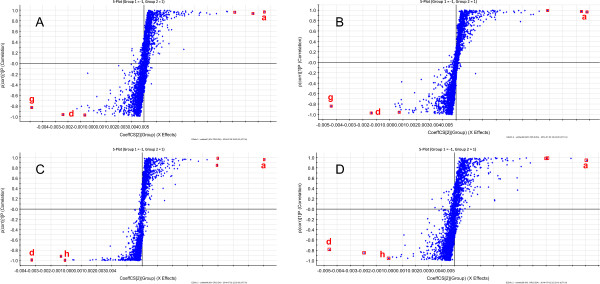
**OPLS-DA(S-plot) of Raw and Processed** ***R. Palmatum*** **Sample: A Raw DH vs. Wine DH; B Raw DH vs. Vinegar DH; C Raw DH vs. Prepared DH; D Raw DH vs. Charred DH.**

Extensive statistical analyses were performed to generate an S-plot, which could be used to identify potential chemical markers for distinguishing between raw and processed *R. palmatum* (Figures 4A and 5, and Table 2). In Figure 4A, the first three ions (i.e., a, b, and c) in the bottom left corner of the “S” are the ions from the raw *R. palmatum* sample that contributed the greatest difference observed in the data between the raw and processed *R. palmatum*. Ions a, b, and c could therefore be used as potential chemical markers to distinguish between samples of raw and processed *R. palmatum*. Ion a was identified as the most characteristic of these three chemical markers in raw *R. palmatum*. Similarly, ions d, e, and f were identified as the most characteristic ions of processed *R. palmatum*, and represented the biggest difference between the raw and processed *R. palmatum*. The ion intensity trends of these ions in the analyzed samples are shown in Figure 4B. Ions a (t_R_ 9.81 min, *m/z* 331.0667, Emodin-8-O-glucoside), b (t_R_ 7.50 min, *m/z* 257.0827, emodin-O-glucoside), and c (t_R_ 7.99 min, *m/z* 451.3296, catechin-glucopyranoside) were detected with higher intensities in the raw samples and lower intensities in the other processed samples. However, these ions were not detected in the prepared *R. palmatum* sample. Ions d (t_R_ 1.43 min, *m/z* 331.0667, Gallic acid-3-O-glucoside), e (t_R_ 9.28 min, *m/z* 245.0824, torachrysone), and f (t_R_10.20 min, m/z Chrysophanol dimethyl ether) were detected with lower intensities in the raw *R. palmatum* sample and higher intensities in the processed samples. Ions d, e, and f could be regarded as potential chemical markers for discriminating between processed and raw *R. palmatum*. Ion d could be used as the most characteristic chemical marker for the identification of processed *R. palmatum*.

**Table 2:** **Marker t**_**R**_ **-m/z ion pairs of raw and processed** ***R***

Raw DH/Processed DH		Raw DH/ vinegar DH		Raw DH/ wine DH		RawDH/ prepared DH		Raw DH/ charred DH	
t_R_- m/z		t_R_- m/z		t_R_- m/z		t_R_- m/z		t_R_- m/z	
Raw DH	9.81-431.0988^(a)^	Raw DH	9.81-431.0988^(a)^	Raw DH	9.81-431.0988^(a)^	Raw DH	9.81-431.0988^(a)^	Raw DH	9.81-431.0988^(a)^
	7.99-451.3296		7.99-451.3296		7.99-451.3296		7.99-451.3296		7.99-451.3296
	3.57-431.0861		3.57-431.0861		3.57-431.0861		3.57-431.0861		3.57-431.0861
Processed DH	1.43- 331.0667^(d)^	Vinegar DH	1.43-331.0667^(d)^	Wine DH	1.43-331.0667^(d)^	Prepared DH	1.43-331.0667^(d)^	Charred DH	1.43-331.0667^(d)^
	9.71- 253.0499		7.33-477.1396^(g)^		7.33-477.1396^(g)^		10.20-281.0814		10.20-281.0841
	10.20-281.0814		8.60-283.0291		8.60-283.0291		9.71- 253.0499^(h)^		9.71-253.0499^(h)^

*Palmatum* samples in the S-plot.

^a^Emodin-8-O-glucoside, ^d^Gallic acid-3-O-glucoside,

^g^Cinnamyl-galloyl-glucoside derivative, ^h^Chrysophanol.

UPLC-QTOF-MS could be used to distinguish raw *R. palmatum* from the different processed *R. palmatum* sampled in conjunction with OPLS-DA (S-plot): Figure 5A raw *R. palmatum* vs. wine *R. palmatum*, Figure 5B raw *R. palmatum* vs. vinegar *R. palmatum*, Figure 5C raw *R. palmatum* vs. prepared *R. palmatum*, and Figure 5D raw *R. palmatum* vs. charred *R. palmatum*. The circled points were regarded as potential characteristic markers that could be used to distinguish raw *R. palmatum* from the different samples of processed *R. palmatum* (Figure 5, Table 2). Ions a (cinnamyl-galloyl-glucoside derivative) and g (t_R_, 8.62 min, *m/z* 461.1085, cinnamyl-galloyl-glucoside derivative) were the identified as the most characteristic chemical markers for distinguishing between raw *R. palmatum* and wine *R. palmatum* (Figure 5A), as well as distinguishing between raw *R. palmatum* and vinegar *R. palmatum* (Figure 5B). Ions a (Emodin-8-O-glucoside) and d (Gallic acid-3-O-glucoside) were determined to be the most characteristic chemical markers for distinguishing between raw *R. palmatum* and prepared *R. palmatum* (Figure 5C), as well differentiating between raw *R. palmatum* and charred *R. palmatum* (Figure 5D). Ions a and d could therefore be used as chemical markers to discriminate between raw and processed *R. palmatum* (including prepared *R. palmatum* and charred *R. palmatum*), whereas ions a and g could be used as the best characteristic markers to distinguish between raw *R. palmatum* and vinegar *R. palmatum*, as well as raw *R. palmatum* and wine *R. palmatum*.

### Preliminary study on processing mechanism

Anthraquinone glycosides make a significant contribution to the hepatic and renal toxicity of raw *R. palmatum*. The Emodin-8-O-glucoside found in *R. palmatum* (ion a) has been reported to be reported to be exhibit the highest levels of hepato- and nephrotoxicity of the anthraquinone glycoside found in *R. palmatum*, whereas chrysophanol (ion h) was reported to be the least toxic of these compounds [26]. The toxicities of the *R. palmatum* materials gradually decreased in the following order: raw *R. palmatum* > wine *R. palmatum* ≈ vinegar *R. palmatum* > prepared *R. palmatum* > charred *R. palmatum* (Table 3). This result may be related to the conversion of the anthraquinone glycosides in the *R. palmatum* samples into the corresponding free anthraquinones in the processed materials. These results provide a better explanation of the previous results (Tables 2 and 3). The cinnamyl-galloyl-glucoside derivative (ion a) was determined to be the most characteristic chemical marker for the identification of raw *R. palmatum*, whereas chrysophanol (ion h) could be used as a potential chemical marker for the identification of prepared and charred *R. palmatum* samples.

**Table 3:** **Toxicity, property and characteristic components of raw and processed DH**

Toxic	Raw DH	Wine DH	Vinegar DH	Prepared DH	Charred DH	References
Nephrotoxicity	√	↓	↓	↓	↓	[16]
Hepatotoxicity	√	↓	↓	↓	↓	[16]
Gastrointestinal reactions	√	↓	↓	↓	↓	[17]
The property of bitter cold	√	↓	↓	↓	↓	[17]
Characteristic components	Aloe-emodin	Cinnamyl-galloyl	Cinnamyl-galloyl	Gallic acid	Gallic acid	[16]
	-glucoside	-glucoside	-glucoside	chrysophanol	chrysophanol	[17]

↓: Reduced toxic effects.

√: Toxic.

Raw *R. palmatum* exhibits a significant heat-clearing effect and purgative activity, which is consistent with the properties of bitter cold and sedimentation described in the theory of TCM. The cinnamyl-galloyl-glucoside derivative (ion a) makes a significant contribution to the purgative effects of *R. palmatum*. Consideration of the purgative effects of the different samples revealed the following trend: raw *R. palmatum* > wine *R. palmatum* ≈ vinegar *R. palmatum* > prepared *R. palmatum* > charred *R. palmatum*. A similar trend was also observed in the bitter cold properties of raw and processed *R. palmatum* samples (Table 3). Gallic acid-3-O-glucoside (ion d) made the greatest contribution to the convergence effect of *R. palmatum*. Consideration of the convergence effects of the different materials revealed the following trend: charred *R. palmatum* > prepared *R. palmatum* > vinegar *R. palmatum* ≈ wine *R. palmatum* > raw *R. palmatum* (Table 3).

Previous studies have indicated that the basic components of raw *R. palmatum* are anthraquinone glycoside compounds, and this information is in agreement with the results of the current study (Tables 2 and 3). Furthermore, the chemical markers of raw and processed *R. palmatum* have been explored in considerable detail in this study. The cinnamyl-galloyl-glucoside derivative was determined to be the most characteristic marker in raw *R. palmatum*, whereas it appeared at a much lower level or even disappeared completely in the prepared and charred *R. palmatum* samples because the anthraquinone glycosides were hydrolyzed to the corresponding anthraquinones during the processing stages, which led to the loss of the purgative activities of these samples and a decrease in their antipyretic effects. Furthermore, the effect of promoting blood circulation to remove blood stasis increased. Gallic acid-3-O-glucoside was identified as the most characteristic marker in the charred and prepared *R. palmatum* samples, and the amount of this material increased significantly in the charred and prepared *R. palmatum* samples compared with the raw material. This result could be attributed to the tannins in the raw material being readily hydrolyzed to form gallic acid-3-O-glucoside by steaming with wine or by carbonization.

## Conclusion

UPLC-QTOF-MS in conjunction with multivariate statistical analysis can be used to screen and identify potential chemical markers that could be used to distinguish raw and processed *R. palmatum.* A variety of different *R. palmatum* samples were processed under the same conditions and six ion pairs were identified as chemical markers that could be used to distinguish the raw materials from that of the processed herbs. Two ions, namely those belonging to the emodin-8-O-glucoside and gallic acid-3-O-glucoside, were determined to be potential chemical markers for processed *R. palmatum.* We have also provided a brief discussion of the underlying mechanisms associated responsible for enhancing the efficacy and reducing the toxicity of *R. palmatum* following the traditional processing procedures. The strategy developed in this study was successfully applied to distinguish between samples of raw from processed *R. palmatum*, and could also be used to investigate the mechanisms responsible for the efficacy enhancing and toxicity reducing effects of crude processing techniques in other materials.

## References

[1] Huang ZS (2002). Traditonal Chinese Medicine.

[2] Jin SY, Wang Q (2004). Studies on Processing of Chinese Medicinal Yinpian and Its Clinical Application.

[3] Cai BC, Gong QF (2009). Processing of Chinese Medicinal Herbs.

[4] China Pharmacopoeia Committee (2010). China Pharmacopoeia.

[5] Kashiwada Y, Nonaka GI, Nishioka I (1989). Studies on Rhubarb (Rhei Rhizoma).XV. Simultaneous determination of phenolic constituents by high-performance liquid chromatography. Cheml Pharm Bull.

[6] Xiao PG, He LY, Wang LW (1984). Studies on the relations of chemical constituents and activities among genus Rheum. J Ethnopharmacol.

[7] Cuellar MJ, Giner RM, Recio MC, Manez S, Rios JL (2001). Topical anti- inflammatory activity of some Asian medicinal plants used in dermatological disorder. Fitoterapia.

[8] Huang Q, Lu G, Shen HM, Chung MC, Ong CN (2007). Anti-cancer properties of anthraquinones from rhubarb. Med Res Rev.

[9] Yokozawa T, Suzuki N, OkudaI Oura H, Nishioka I (1985). Changes in the uri-nary constituents in rats with chronic renal failure during oral administration of rhubarb extract. Chem Pharml Bull.

[10] Zhao YL, Wang JB, Zhou GD, Shan LM, Xiao XH (2009). Investigations of free anthraquinones from rhubarb against α-naphthylisothiocyan- ate-induced cholestatic liver injury in rats. Basic Clinl Pharmacol.

[11] Zhao GP, Dai S, Chen RS (2006). Dictionary of Traditional Chinese Medicine.

[12] World Health Organization (2002). WHO monographs on selected medicinal plants (Vol. 2).

[13] Wang CF, Wu XD, Chen M, Duan WG, Sun LX, Yan M, Zhang LY (2007). Emodin induces apoptosis through caspase 3-dependent pathway in HK-2 cells. Toxicology.

[14] Zhang LY, Jiang ZZ, Pu CH, Yan M (2004). Six-month oral toxicity study of total anthraquinone in Radix et Rhizoma Rhei in SD rats. Chin J Biochem Pharm.

[15] Wang JB, Zhao YL, Xiao XH, Li HF, Zhao HP, Zhang P, Jin C (2009). Assessment of the renal protection and hepatotoxicity of rhubarb extract in rats. J Ethnopharmacol.

[16] Wang JB, Ma YG, Zhang P, Jin C, Sun YQ, Xiao XH, Zhao YL, Zhuo C-P (2009). Effect of processing on the chemic al contents and hepatic and renal toxicity of rhubarb studied by canonical correlation analysis. Acta Pharm Sin.

[17] Guo P, Zhang TJ, Zhu XY, He YZ (2009). Study on toxicity of Radixet Rhizoma Rhei and countermeasure for its attenuation. Chin Tradit Herb Drugs.

[18] Drug administration in Ministry of Health (1988). Prepared standard of traditional chinese medicine in China.

[19] Churchwella MI, Twaddlea NR, Meekerb CL, Doergea DR (2005). Improving LC-MS sensitivity through increases in chromatographic performance: comparisons of UPLC-ES/MS/MS to HPLC-ES/MS/MS. J Chromatogr B.

[20] Jiang X, Huang LF, Wu LB, Wang ZH, Chen SL (2012). UPLC-QTOF/MS analysis of Alkaloids in Traditional Processed Coptis chinensis Franch. Evid-Based Complement Altern Med.

[21] Wu LB, Jiang X, Huang LF, Chen SL (2013). Processing Technology Investigation of Loquat (Eriobotrya japonica) Leaf by Ultra-Performa- nce Liquid Chromatography-Quadrupole Time-of-Flight Mass Spectrometry Combined with Chemometrics. PLoS One.

[22] Jiang HQ, Rong R, Lu QT (2011). Identification of chemical composition in Rhunarb by high performance Liquid Chromatography with Mass Spectrometry. LiShiZhen Med Materia Med Res.

[23] Ye M, Han J, Chen HB, Zheng JH, Guo D (2007). Analysis of phenolic compounds in rhubarbs using liquid chromatography coupled with electrospray Ionization mass spectrometry. J Am Soc Mass Spectr.

[24] Sun H, Zhu C, Zhang HY, Wang YR, Luo GA, Hu P (2009). Comparative analysis of main constituents of Radix et Rhizoma Rhei and processed Radix et Rhizoma Rhei by HPLC-ESI-TOF-MS. Chin Tradit Patent Med.

[25] Ma XH, Shen S, Han FM, Chen Y (2006). The electrospray ionization-mass spectra of Radix et rhizoma rhei anthraquinones. Journal of Hubei University (Natural Science).

[26] Wiklund S, Johansson E, Sjöström L, Mellerowicz E-J, Edlund U, Shockcor J-P, Gottfries J, Moritz T, Trygg J (2008). Visua lization of GC/TOF-MS-based metabolomics data for identification of biochemically interesting compounds using OPLS class models. Anal Chem.

[CR27] The pre-publication history for this paper can be accessed here:http://www.biomedcentral.com/1472-6882/14/302/prepub

